# Noninvasive Imaging for Patients with COVID-19 and Acute Chest Pain

**DOI:** 10.14797/mdcvj.1040

**Published:** 2021-12-15

**Authors:** Awad Javaid, Yehia Saleh, Ahmed Ibrahim Ahmed, Jean Michel Saad, Maan Malahfji, Mouaz H. Al-Mallah

**Affiliations:** 1 Kirk Kerkorian School of Medicine at the University of Nevada Las Vegas School of Medicine, Las Vegas, NV, US; 2 Houston Methodist DeBakey Heart & Vascular Center, Houston, TX, US

**Keywords:** COVID-19, coronavirus, cardiovascular disease, acute coronary syndromes, noninvasive imaging

## Abstract

Acute chest pain is a common presentation in patients with COVID-19. Although noninvasive cardiac imaging modalities continue to be important cornerstones of management, the pandemic has brought forth difficult and unprecedented challenges in the provision of timely care while ensuring the safety of patients and providers. Clinical practice has adapted to these challenges, with several recommendations and societal guidelines emerging on the appropriate use of imaging modalities. In this review, we summarize the current evidence base on the use of noninvasive cardiac imaging modalities in COVID-19 patients with acute chest pain, with a focus on acute coronary syndromes.

## Introduction

The coronavirus disease 2019 (COVID-19) pandemic, caused by severe acute respiratory syndrome coronavirus 2 (SARS-CoV-2), continues to cause significant morbidity and mortality.^1^ As of December 1, 2021, approximately 71.3% of US adults were fully vaccinated, yet outbreaks continue due to the highly transmissible Delta variant and low vaccination rates in certain areas of the country.^2^ Furthermore, vaccine inequity and the emergence of highly transmissible variants have been the main drivers for the resurgence of cases in developing nations.^3^ In recent months, the significant increase in pediatric cases has also fueled the continued spread of the virus.^4^

Acute chest pain is a common presentation in patients with COVID-19. Although the true prevalence is unknown, studies have shown rates as high as 40% to 60%.^5,6^ Accurate diagnosis can be frustrating to clinicians because the etiology can be due to several different pathophysiologic processes (***Table 1***).

**Table 1:** Differential diagnosis of chest pain in patients with COVID-19. ECG: electrocardiogram; STEMI: ST-elevation myocardial infarction; NSTEMI: non-ST-elevation myocardial infarction; ICA: invasive coronary angiogram; CAD: coronary artery disease; TTE: transthoracic echocardiogram; POCUS: point-of-care ultrasound; CCTA: coronary computed tomography angiography; JVP: jugular venous pressure; COVID-19: coronavirus disease 2019; PNA: pneumonia; CT: computed tomography; CTPA: computed tomography pulmonary angiography; LV: left ventricle; RWMA: regional wall motion abnormalities; MINOCA: myocardial infarction with nonobstructive coronary arteries; CMR: cardiac magnetic resonance imaging; LGE: late gadolinium enhancement; DVT: deep vein thrombosis; ACS: acute coronary syndrome


DISEASE PROCESS	PRESENTATION	IMAGING FOR INITIAL AND SUBSEQUENT ASSESSMENT

Acute coronary syndrome	Ischemic chest pain with typical ECG changes and elevation of cardiac biomarkers	STEMI-ICA to evaluate for obstructive CAD NSTEMI/unstable angina: urgent TTE or POCUS followed by CCTA in low risk or ICA if high risk

Cardiac tamponade	Dyspnea, tachypnea, elevated JVP, hypotension, pulsus paradoxus, electrical alternans on ECG	Stat TTE to evaluate for large pericardial effusion with chamber collapse/respiratory variation in volumes and flows

COVID-19 pneumonia	Cough, fever, myalgia, headache, dyspnea, loss of smell/taste, worsening hypoxemia	CT to simultaneously evaluate for PNA (chest CT), PE (CTPA), and obstructive CAD (CCTA), depending on suspicion

Heart failure, new onset, without hypotension	Progressive dyspnea, signs of congestion, S3 heart sound	TTE to evaluate LV function If RWMA present and suspicion of chronic ischemia, consider CCTA in lower risk or ICA in higher risk

MINOCA	Ischemic chest pain with ECG changes and modest elevation of cardiac biomarkers	ICA shows no obstructive CAD CMR to evaluate for subendocardial or transmural pattern corresponding to vascular territory

Myocarditis	Variable but may include presentation of acute heart failure with new arrhythmias	CMR to evaluate for LGE with myocardial edema

Pericarditis	Pleuritic chest pain, diffuse ST elevations and PR depressions on ECG, pericardial friction rub	TTE to evaluate for pericardial effusion

Pulmonary embolism	Dyspnea, pleuritic chest pain, signs and symptoms of DVT	CT to simultaneously evaluate for PNA (chest CT), PE (CTPA), and obstructive CAD (CCTA), depending on suspicion

Stress cardiomyopathy	Similar to ACS with signs of heart failure	ICA to evaluate for obstructive CAD TTE to evaluate for wall motion abnormalities


## Epidemiology of Acute Coronary Syndromes

Acute coronary syndromes (ACS) are one such group of diseases requiring urgent diagnosis and management. Patients with COVID-19, particularly those with cardiovascular risk factors, have an increased risk of ACS. Studies have shown several mechanisms of cardiovascular injury, such as hypoxemia, hypercoagulability, cytokine storm, and inflammatory myocarditis.^7^

Much like its profound effect on the discipline of medicine, the pandemic has altered the epidemiology of ACS. Several studies have reported a decline in the incidence of emergency room visits for chest pain and hospitalizations for acute myocardial infarction.^8,9,10^ A similar decline was seen in the rates of referral for percutaneous coronary intervention.^11,12^ Some have posited that this may be due to a change in health-seeking behavior because patients may be more cautious about going to the emergency department (ED) for fear of exposure to COVID-19. However, another study showing a similar decrease in acute myocardial infarction (MI) presentations without a reduction in out-of-hospital cardiac arrests indicates that there may actually be a decrease in the incidence of acute MI.^13^

Noninvasive cardiac imaging plays an important role in the diagnosis and management of patients presenting with acute chest pain. As clinical practice adapts to the pandemic, several recommendations and societal guidelines have been published to optimize the management of acute chest pain in patients with COVID-19 while ensuring the safety of providers.

## Global Trends in Noninvasive Cardiac Imaging during the Pandemic

There has been a global decline in the volume of cardiovascular imaging during the pandemic. Testing across all modalities decreased by an average of 40% to 60% in 2020. Rates differed by modality, with a reduction of 54% for coronary computed tomography angiography (CCTA), 59% for echocardiography, and 78% for stress testing.^14^ Reductions were similar in the US, with higher rates in the Midwest.^15^

## ST-Elevation Myocardial Infarction

Urgent diagnosis and treatment are essential for the survival and long-term prognosis of patients with ST-elevation MI (STEMI), irrespective of COVID-19 status. This is even more important considering studies that have shown higher rates of cardiogenic shock, recurrent MI, stent thrombosis, repeat revascularization, and in-hospital mortality in such patients.^16,17,18,19^ Studies during the early period of the outbreak showed lower rates of invasive angiography, delays in symptom-to-admission, and prolonged door-to-balloon time, all of which potentially contributed to sub-par management and worse outcomes.^20,21,22,23^

Considering the urgency in STEMI diagnosis and management, noninvasive imaging has a limited role in STEMI patients with COVID-19. A recent joint position statement from the Society for Cardiovascular Angiography and Intervention (SCAI) and the American College of Cardiology (ACC) recommend primary percutaneous coronary intervention in COVID-19 patients presenting with STEMI provided that there is adequate personal protective equipment for staff and a dedicated catheterization lab.^24^

## Non–ST-Elevation ACS and Other Causes of Chest Pain

Noninvasive imaging modalities have a broader role in COVID-19 patients with non–ST-elevation ACS. Patients with COVID-19 are at higher risk of non-ST-elevation ACS. CCTA, echocardiography, and cardiac magnetic resonance imaging (CMR) have been suggested to play a role in such patients.

### Coronary Computed Tomography Angiography

**Figure 1:**
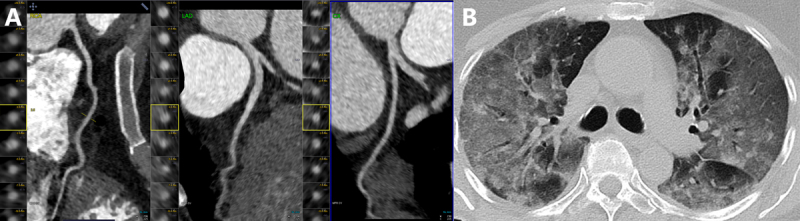
Coronary computed tomography (CT) angiography in a 34-year-old woman who presented with COVID-19 pneumonia. The patient had chest pain and minimal troponin elevation. **(A)** Coronaries were free of atherosclerosis or stenosis. **(B)** Chest CT shows advanced COVID-19 pneumonia.

CCTA has gained popularity over the last decade due to its high negative predictive value in the evaluation of both stable and acute chest pain.^25,26^ Considering the ubiquitous use of chest CT in COVID-19 patients, CCTA can be done in the same session (***Figure 1***). Many hospitals have seen a substantial increase in the use of CCTA for evaluating acute chest pain.^27^ In the ED setting, CCTA has been effective in expediting care for patients with acute chest pain and helping to avoid inpatient admissions for hospitals operating near full capacity. A study of 513 patients at a tertiary care referral hospital in Dublin, Ireland, showed that CCTA use more than doubled after COVID-19 lockdown was instituted and was associated with a significantly reduced rate of invasive coronary angiography (ICA) and patient length of stay without an increase in 30-day adverse outcomes.^28^

In March 2020, the Society of Cardiovascular Computed Tomography (SCCT) released guidance for CCTA in COVID-19 patients that was endorsed by the ACC. The document emphasized the use of CCTA for assessment of acute chest pain with sufficient suspicion for CAD and high-risk stable chest pain; it also recommended postponing imaging for up to 4 or more weeks in patients who have stable chest pain without high suspicion for CAD.^29^

One of the most advantageous roles of CCTA during the COVID-19 pandemic has been in helping to determine the etiology of elevated troponin levels for patients in whom STEMI was ruled out.^27^ CCTA identifies patients with CAD who can be treated conservatively by excluding high-risk anatomies and physiologically significant lesions with fractional flow reserve CT.^30^ A consensus statement by SCAI, the ACC, and the American College of Emergency Physicians on the management of acute MI during the COVID-19 pandemic says that CCTA may be considered in cases where the findings of ST-elevation and transthoracic echocardiography are divergent.^24^ The use of CCTA in such cases would help reduce the rates of unnecessary ICA. Delayed iodine-enhanced CCTA is useful to differentiate MI from other etiologies in patients with MI with nonobstructive coronary arteries (MINOCA) and to evaluate patients with suspected myocarditis.

In lower-risk patients with equivocal ECG changes and possible non-STEMI or unstable angina, CCTA can be a helpful tool for further risk stratification. For patients with high-risk ACS, CCTA is not preferred because these patients with high pre-test probability of obstructive CAD should undergo ICA, which can offer time-sensitive interventions. In addition, CCTA is less preferred than CMR for MINOCA after ICA because it exposes patients to radiation and cannot simultaneously exclude multiple etiologies.^31^

### Echocardiography

Of the COVID-19 patients without pre-existing cardiac disease who undergo echocardiography, nearly half have abnormal findings.^16^ Echocardiography is usually the first cardiac imaging modality to be employed in the evaluation of acute chest pain for patients with COVID-19. Point-of-care ultrasound (POCUS) in the hands of an experienced operator can accelerate the time taken to make a diagnosis. It is cost-effective, portable, and aids in selecting patients for more advanced echocardiographic imaging. Patients with an equivocal diagnosis of STEMI or acute chest pain with suspected ACS should undergo POCUS to assess for wall motion abnormalities.^17,24^ Urgent echocardiography and/or POCUS can quickly assist in triaging patients and aid in determining the next imaging tool for evaluation, whether it be formal echocardiography, CCTA, ICA, or chest CT. However, POCUS images are less accurate than transthoracic echocardiography (TTE) and represent only gross estimations of ejection fraction, regional wall motion abnormalities, and valvular disease. Formal TTE can obtain more accurate and detailed images but is usually not feasible in an emergent situation such as STEMI.

Echocardiography should be used to assess for left ventricular (LV) and right ventricular (RV) function, valvular abnormalities, wall motion abnormalities, and pericardial effusion. Because there is considerable overlap in clinical presentation between cardiac and pulmonary complications in COVID-19, echocardiography has an important role in differentiating the two. SARS-CoV-2 commonly induces a prothrombotic state, and pulmonary embolism (PE) is a common complication.^18^ Echocardiography can rapidly identify RV strain in patients with PE and help them get appropriate further treatment. Right ventricular function has been found to be a particularly important prognostic factor. In a study of 510 patients with COVID-19, RV dysfunction and dilation each conferred increased mortality risk.^19^ Right ventricular remodeling conferred a greater than 2-fold increase in mortality risk when controlled for age and biomarker elevation, and patients without adverse RV remodeling were more likely to survive until hospital discharge.

An important principle in the use of echocardiography is that it must only be performed in situations that will provide net clinical benefit to minimize infection risk to staff.^32,33^

### Cardiac Magnetic Resonance Imaging

Since the beginning of the pandemic, CMR has proven to be valuable in differentiating various COVID-19–related disease processes. Myocardial injury is common in COVID-19 and associated with higher mortality.^34^ Acute myocarditis can present with chest pain and/or shortness of breath, which can mimic both ACS and exacerbation of chronic LV dysfunction. In equivocal cases with troponin elevation, CMR can evaluate ventricular function, myocardial ischemia, myocardial tissue viability, and valvular function all at once with limited exposure to infection. In cases of MINOCA, the presence of myocardial inflammation or edema on CMR can help distinguish acute from chronic LV dysfunction.

In select patients with COVID-19, CMR is considered the gold standard to evaluate for acute myocarditis when the clinical presentation and biomarkers suggest acute myocardial inflammation and other relevant conditions such as ACS and heart failure are ruled out. CMR was used in three case series to diagnose myocarditis temporally associated with vaccination for COVID-19 and no other risk factors.^35,36,37,38^ In such cases, CMR is useful because it can reveal diffuse myocardial edema causing pseudo-wall hypertrophy, noninfarct patterns of late gadolinium enhancement, and increased signal on short T1 inversion recovery, T1 mapping, and T2 mapping sequences.^39,40^ As the pandemic has progressed, a larger portion of the population has been vaccinated, and COVID-19–vaccine-induced myocarditis has been recognized as a distinct phenomenon, for which CMR has been indispensable.^41^

Although patients with COVID-19 have been presenting with signs or symptoms of myocarditis-like disease throughout the pandemic, it should be noted that recent studies have called into question whether these presentations can be categorized as classic myocarditis. In an editorial by Sengupta and Chandrashekhar, the authors cited the lack of consistent lymphocytic infiltration in endomyocardial biopsies and cautioned clinicians to be more careful in using the term myocarditis for COVID-19 patients.^42^ Regardless, for patients with acute chest pain in which STEMI has been ruled out, CMR remains a valuable tool to differentiate multiple disease states.

The Society for Cardiovascular Magnetic Resonance released a guidance document in 2020 for the practice of CMR during the pandemic. The guideline stressed that, as with other imaging modalities, studies should be deferred to the outpatient setting and postponed until recovery from acute illness unless findings are likely to impact acute management.^43^ Also highlighted was the crucial role of CMR in differentiating myocardial injury and the importance of personal protective equipment (PPE). To minimize infection risk, shortened and focused imaging protocols less than 30 minutes should be used when possible and only when they will likely alter management.^40,43^

The main limitation of CMR is its lack of widespread availability. Furthermore, its longer acquisition time restricts its use in certain situations. For example, in patients with severe COVID-19 pneumonia and elevated cardiac biomarkers who are being evaluated for obstructive CAD and PE, CCTA can be used alongside CT of the chest to quickly reach a diagnosis.

### Nuclear Imaging

**Figure 2:**
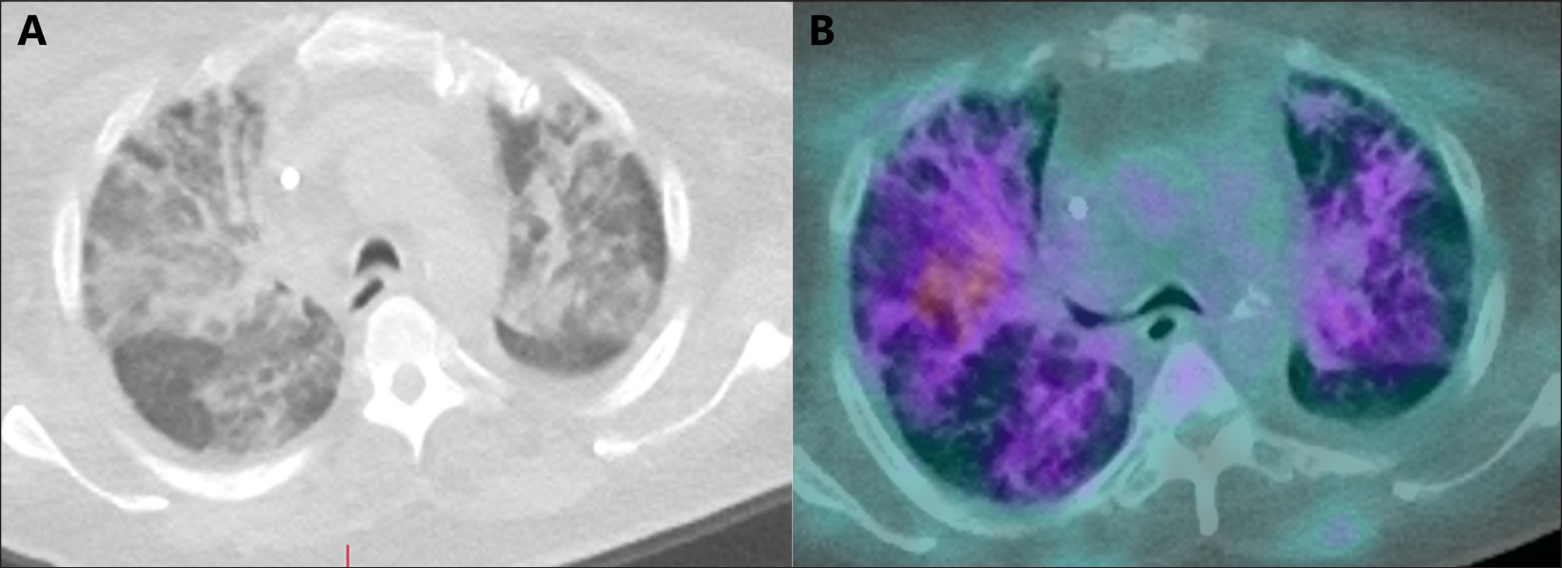
**(A)** Severe pulmonary infiltrates in a patient with recent COVID-19 infection referred for positive emission tomography myocardial perfusion imaging. **(B)** There is rubidium uptake in these pulmonary infiltrates.

Nuclear cardiology plays an important role in the evaluation of patients with suspected or known CAD (***Figure 2***).^44,45,46^ One of the first societal guidelines regarding imaging practices during COVID-19 was jointly published by the American Society for Nuclear Cardiology and the Society for Nuclear Medicine and Molecular Imaging.^47^ The document set forth best practices for the evaluation of all patients, including those presenting with emergent conditions, largely based on general principles of infection prevention and expert opinion.

Of relevance to patients with acute chest pain, myocardial perfusion imaging (MPI) was categorized as a high-priority study to be performed with no postponement in intermediate- to high-risk ACS patients being considered for urgent revascularization and intermediate-risk patients with CAD symptoms but at high risk for ICA. Furthermore, regadenoson is preferred to adenosine or dipyridamole considering the short single infusion that is required. Lastly, the guideline suggests evaluating the lungs for possible pulmonary findings using hybrid single-photon emission computerized tomography (SPECT) and positron emission tomography (PET)-CT systems with attenuation correction. Such incidental findings have been demonstrated in other settings, such as patients with suspected CAD and those undergoing fluorodeoxyglucose-PET for oncologic or neurologic indications.^48,49,50^

As with other modalities, the rates of nuclear cardiology testing have decreased during the COVID-19 pandemic, with several studies reporting decreases ranging from 50% to 90%. However, at least two studies showed that the rates of abnormal SPECT findings have remained the same, suggesting that high-risk patients were either not prioritized or missed altogether.^51,52^ Even though other imaging modalities were used more often during the pandemic, a recent study of patients with SPECT MPI testing after hospitalization for COVID-19 found no patient who experienced imaging-related adverse events. Even though this was a small cohort (n = 15) and none had prior MI, chest pain was the indication for imaging in 50% of these patients.^53^

One of the main drawbacks of nuclear imaging is acquisition time, which is an important factor during the pandemic given that minimizing infection risk is a high priority. Also, nuclear imaging has a curbed role in acute clinical scenarios. Regardless, nuclear imaging can still be used to evaluate myocardial viability or identify patients with implanted device infection as well as for patients with coronary stents, significant coronary calcification, dye allergy, and reduced renal function.^30^

## General Considerations

### Mode of Stress Testing

Exercise stress testing is one of the mainstays in the evaluation of suspected CAD. For acute chest pain, exercise stress testing can be beneficial in those with borderline or negative serial ECG and troponin with low-risk non-STEMI or unstable angina, per the appropriate utilization guidelines.^54^ At the same time, exercise stress testing is considered an aerosol-generating procedure that significantly increases the risk of transmission to providers. Thus, guidelines recommend using pharmacologic stress testing with vasodilators to minimize droplet exposure.^55^ During the COVID-19 pandemic, a consistent theme in society guidelines has been the recommendation to minimize aerosolization risk from imaging procedures. In patients with COVID-19, CCTA is preferred to stress testing for evaluation of low- to intermediate-risk of CAD to minimize aerosolization risk of exercise testing.^30^ Accordingly, the number of stress tests performed has dropped significantly in many centers in favor of CCTA.^27^ Exercise echocardiography should generally be avoided due to the risk of aerosolization.^17^ As the pandemic recedes in many parts of the US, exercise stress testing will continue to increase; however, providers should continue to be cognizant of infection risk. Clinicians should know air circulation patterns in the testing environment, allow for air changes before bringing new patients into the room, avoid manual blood pressure measurement, maintain 6 feet of distance from patients, and don appropriate PPE.^30^

### PPE and Infection Prevention

A persistent problem during the early period of the pandemic was a lack of PPE. Many healthcare systems were ill-prepared for the ever-growing need for PPE, which put providers at risk of infection and increased the spread of infection within healthcare settings. Modalities such as echocardiography and exercise stress testing posed a higher risk due to their aerosol-generating nature.

All major guidelines on cardiovascular imaging advocated for the proper use of PPE.^29,43,47,56^ Suggestions included standard (handwashing and the use of gloves), droplet (head cover, face mask, and eye shield), and airborne (N-95/N-99 masks, respirators) precautions. Although the level of PPE use depends on the disease status of the patient, many guidelines recommended airborne precautions even for suspected cases.

Further recommendations involve cleaning scanners and equipment before and after each patient, regularly disinfecting air-conditioning systems, and fumigating the facility. Some also suggested dedicating one scanner for COVID-19–positive patients when possible.

Guidelines also suggested opportunities to minimize patient-provider contact, such as remote patient screening, a preference for verbal or minimized written consent, and the use of telehealth to communicate results. Similarly, rapid imaging protocols were recommended to reduce opportunities for transmission.

## Novel Applications of Imaging

Noninvasive imaging modalities also have had novel applications in patients with COVID-19. Nuclear imaging modalities have been shown to detect inflammation associated with COVID-19. 2-[18F]fluoro-2-deoxyglucose PET has been used to visualize inflammatory cells in the lungs of asymptomatic and symptomatic patients infected with SARS-CoV-2.^57,58^ Molecules that selectively bind to infected cells or structural proteins can potentially be used to visualize active infection, as has been demonstrated in imaging of herpes simplex infection.^59,60^ Furthermore, one study has shown how radionuclides can be used to target therapy to SARS-CoV-2 virion or infected cells.^61^

## Multimodality Imaging

**Figure 3:**
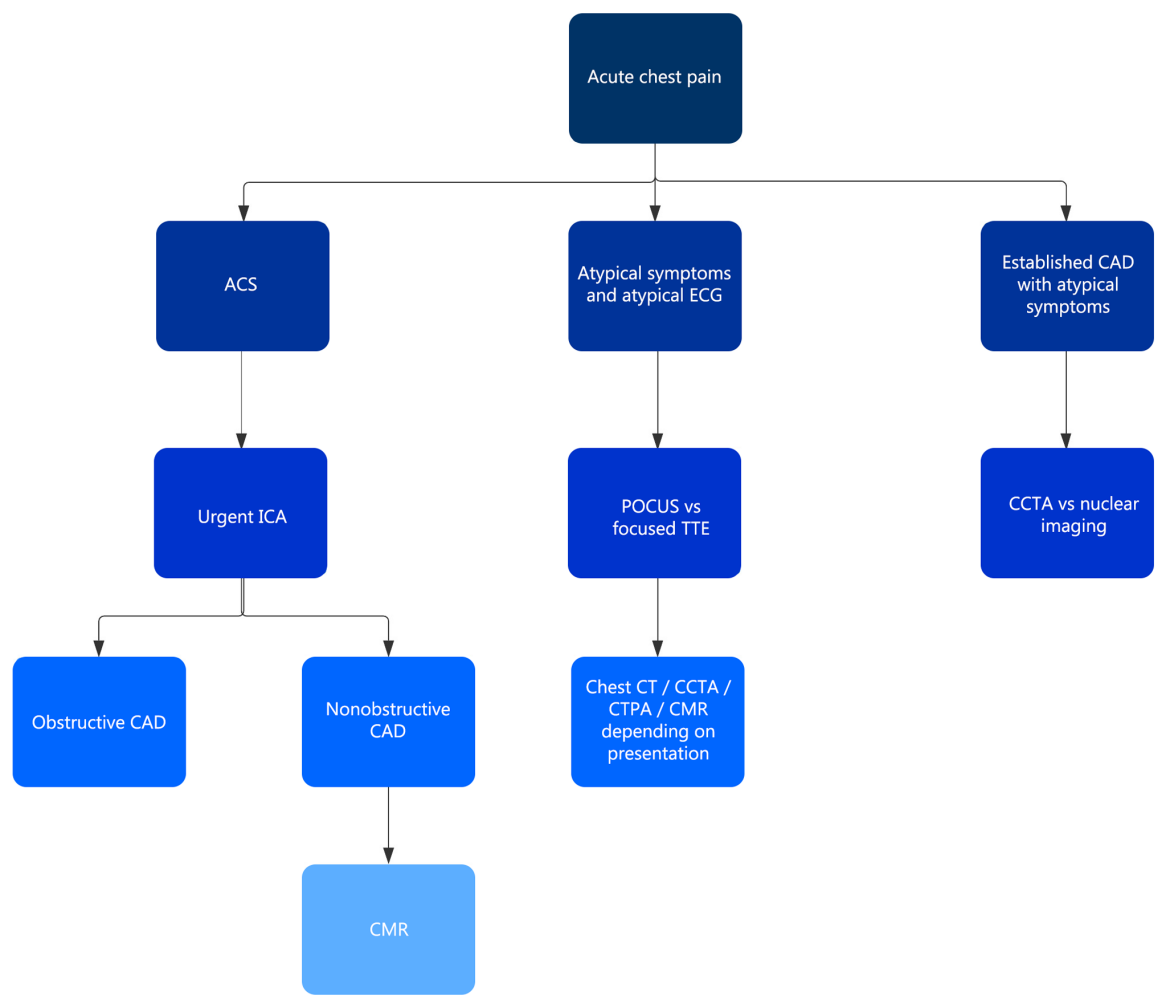
Flowchart for noninvasive imaging in patients with COVID-19 presenting with acute chest pain. ACS: acute coronary syndrome; ECG: electrocardiogram; CAD: coronary artery disease; ICA: invasive coronary angiography; POCUS: point-of-care ultrasound; TTE: transthoracic echocardiogram; CCTA: coronary computed tomography angiography; CT: computed tomography; CTPA: computed tomography pulmonary angiography; CMR: cardiac magnetic resonance imaging

Because the presentation of chest pain in patients with COVID-19 has a broad differential diagnosis, the employment of multiple imaging techniques for a specific case is a critical theme. A patient admitted with COVID-19 pneumonia with elevated cardiac biomarkers and suspected heart failure may undergo a focused TTE or POCUS to evaluate LV function in the ED, CCTA to anatomically evaluate for obstructive CAD, CMR to rule out alternate diagnoses, and nuclear imaging to functionally evaluate for obstructive CAD later in the hospitalization. A team-based approach with staff from each imaging department working together can quickly and accurately provide patients with a diagnosis in a scenario that may initially seem difficult to evaluate. An imaging algorithm for patients with COVID-19 and acute chest pain is displayed in ***Figure 3***.

## Conclusion

Although vaccines and public health measures have helped reduce COVID-19 morbidity and mortality, the emergence of more virulent strains and vaccine inequity means that it will take time before clinical practice can return to a prepandemic state. With adherence to patient-focused evaluations in addition to general infection prevention measures, imaging techniques can continue to provide important diagnostic and prognostic information in patients with acute chest pain, ultimately contributing to improved patient care.

## Key Points

- Acute chest pain is a common presentation in patients with COVID-19.
- The role of noninvasive cardiac imaging is limited in COVID-19 patients with ST elevation myocardial infarction because priority is immediate transfer to the catherization lab.
- Coronary computed tomography angiography, echocardiography, and cardiac magnetic resonance imaging play an important role in patients with non–ST-elevation acute coronary syndromes.
- Cardiac imaging modalities must only be employed if it will help narrow down the diagnosis or change impending management.
- Due consideration should be given to avoiding aerosol-generating procedures, providing adequate personal protective equipment to providers, and streamlining the imaging encounter.
